# A bimetallic Fe–Mg MOF with a dual role as an electrode in asymmetric supercapacitors and an efficient electrocatalyst for hydrogen evolution reaction (HER)†

**DOI:** 10.1039/d3ra04279k

**Published:** 2023-09-05

**Authors:** Asma Zaka, Muhammad Waqas Iqbal, Amir Muhammad Afzal, Haseebul Hassan, Hira Rafique, Saikh Mohammad Wabaidur, Ahmed M. Tawfeek, Eshan Elahi

**Affiliations:** a Department of Physics, Riphah International University, Campus Lahore Pakistan; b Chemistry Department, College of Science, King Saud University Riyadh 11451 Saudi Arabia; c Department of Physics, Sejong University Republic of Korea waqas.iqbal@riphah.edu.pk

## Abstract

In this work, a novel bimetallic Fe–Mg/MOF was synthesized through a cost-effective and rapid hydrothermal process. The structure, morphology, and composition were examined using X-ray diffraction (XRD), scanning electron microscopy (SEM), and X-ray photoelectron spectroscopy. Further, the Brunauer–Emmett–Teller (BET) measurement showed a 324 m^2^ g^−1^ surface area for Fe–Mg/MOF. The Fe–Mg/MOF achieved 1825 C g^−1^ capacity at 1.2 A g^−1^ current density, which is higher than simple Fe-MOF (1144 C g^−1^) and Mg-MOF (1401 C g^−1^). To assess the long-term stability of the asymmetric device, the bimetallic MOF supercapattery underwent 1000 charge/discharge cycles and retained 85% of its initial capacity. The energy and power densities were calculated to be 57 W h kg^−1^ and 2393 W kg^−1^, respectively. Additionally, Fe–Mg/MOF showed superior electrocatalytic performance in hydrogen evolution reaction (HER) by demonstrating a smaller Tafel slope of 51.43 mV dec^−1^. Our research lays the foundation for enhancing the efficiency of energy storage technologies, paving the way for more sustainable and robust energy solutions.

## Abstract 2

A novel energy storage device that exhibits superior power and energy density compared to traditional supercapacitors and batteries.

## Graphical abstract

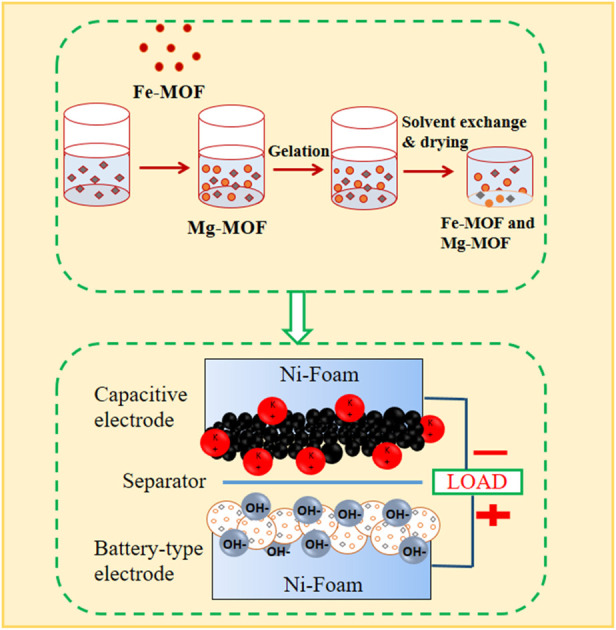

## 1. Introduction

The increased awareness of environmental concerns, as well as the increasing demand for advanced power solutions in electric vehicles and smart grids, have resulted in an urgent need to enhance energy storage systems and technologies. Supercapacitors (SCs) have emerged as an up-and-coming alternative owing to their exceptional qualities, encompassing high power density, extended lifespan, and rapid charging capabilities.^1–4^ These advantageous characteristics make them an attractive choice for various applications in energy storage. Still, supercapacitors' relatively low energy density presents a hurdle to their extensive utilization in areas that simultaneously fulfil high energy and power requirements. Conventional electrical double-layer capacitors (EDLCs) rely on an electrostatic mechanism, storing charge through rapid ion adsorption. However, this approach limits their capacitance due to the inherent nature of the process. Hybrid supercapacitors have recently gained considerable interest as an alternative to EDLCs. A carbon-based capacitive electrode is combined with an alternative battery-type electrode made of transition metals to produce supercapacitors. The key factors driving this attention are their enhanced capacitance and expanded operating voltage range.^5–9^

On the other hand, incorporating a transition metal-based electrode into hybrid supercapacitors can lead to certain drawbacks that may affect the cycling stability, an essential characteristic of EDLCs.^10–14^ Metal–organic frameworks have attracted substantial attention as electrode materials for energy storage and conversion because of their remarkable flexibility to change their structure and excellent porosity. These materials are gaining increasing interest in various energy-related applications.^15–24^ While some of the previously mentioned metal–organic frameworks (MOFs) or MOF-derived materials can attain high operating voltage, their practical application is still limited due to their relatively low capacitance.^25^ Recent studies have highlighted the potential of MOF-74-M (where M represents various metal ions like Mg^2+^, Mn^2+^, Ni^2+^, Co^2+^, Zn^2+^, *etc.*) as a valuable material used in synthesising composite materials. This is primarily attributed to the potential existence of one or more metal centres within MOF-74-M.^26,27^ Furthermore, a recent development involves the direct carbonization of bimetallic MOF (iron-MOF, magnesium MOF and iron-magnesium-MOF), resulting in synthesised mixed iron and magnesium oxides embedded within a nono-porous carbon framework. Although there have been previous reports on bimetallic MOF-derived hybrid materials, these materials still exhibit certain drawbacks when utilized as electrodes for supercapacitors.^28,29^

This study aims to introduce a novel class of hybrid materials, Fe-MOF, Mg-MOF, and Fe–Mg/MOF which are named as FM, MM, and MFM, respectively. To our knowledge, no previous reports have been on synthesizing these hybrid materials using bimetallic MFM. When employed as supercapacitor electrodes, the MFM demonstrates exceptional performance, with an impressive specific capacity of 1825 C g^−1^ and a current density of 1.2 A g^−1^. The MFM along activated carbon set in a two cell assembly for real device fabrication. The MFM//AC device achieves a high energy density of 57 W h kg^−1^ at a power density of 2393 W kg^−1^. These findings emphasize the immense potential of MFM in enabling excellent energy storage devices.

## 2. Experimental

### 2.1. Chemicals

The substances used in this study were sourced from Sigma Aldrich Japan. This includes MOF-74 with the dobdc^4−^ ligand (H4dobdc = 2,5-dihydroxyterephthalic acid 1,4-benzenedicarboxylic acid), iron(iii) chloride hexahydrate (FeCl_3_·6H_2_O), and *N*,*N*′-dimethylformamide (DMF). We obtained 2,5-dihydroxyterephthalic acid (H_4_DOBDC) from the appreciated Tokyo Chemical Industry company, while methanol (CH_4_O) and polyvinylpyrrolidone (PVDF) were purchased from Nacalai Tesque. The Mg (MgCl_2_6H_2_O), dimethylacetamide (DMAc), HCl, and KOH were provided by Sinopharm Chemical Reagent Co., whereas Aladdin Co. supplied 1,3,5-benzenetricarboxylic acid and 1,4-benzenedicarboxylic acid, both with a purity of 95%. All chemicals were used in their as-received without undergoing any further purification procedures.

### 2.2. Preparation of MOFs

**Fig. 1:**
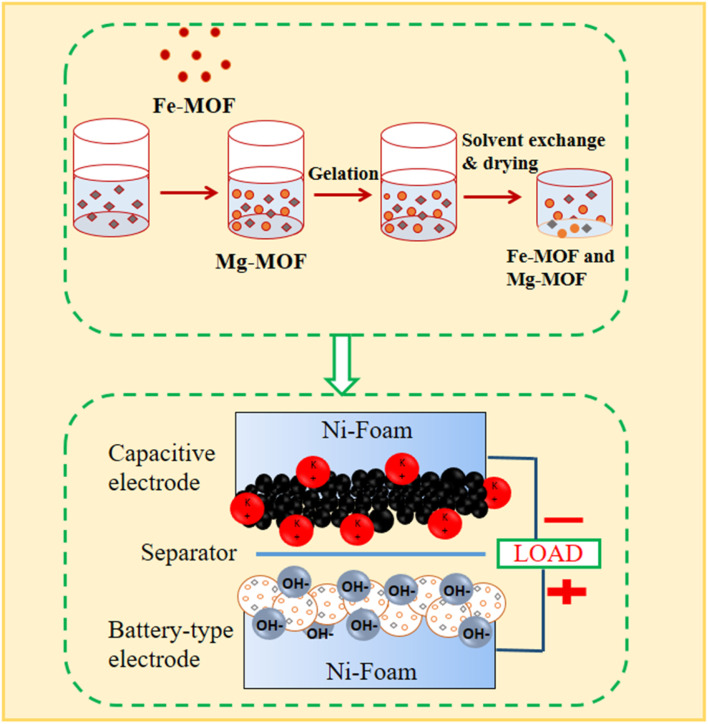
A precise schematic illustration of the metal–organic framework (MOF) synthesis method.

Using a previously described technique, Fe^3+^ : Mg^2+^ bimetallic organic frameworks with different molar ratios (0.6 : 1 and 1 : 1) were prepared^27^ as shown in Fig. 1. We made a solution by mixing a 1 mmol of a solution of magnesium nitrate hexahydrate (Mg(NO_3_)_2_·6H_2_O) and 1 mmol of iron(iii) nitrates nonahydrate (Fe(NO_3_)_3_·9H_2_O), and 1 mmol of terephthalic acid (H_2_BDC) into 10 mL of *N*,*N*′-dimethylformamide. The mixture was thoroughly mixed before being heated for 8 hours at 120 °C in an autoclave. The solid particles were separated by centrifugation after the process, after which the resultant mixture was cooled to room temperature. The combined particles were repeatedly cleaned in *N*,*N*′-dimethylformamide (DMF) before being vacuum-dried at 120 °C. The same method was used to individually synthesize monometallic compounds based on FM and MM, utilizing only the metal precursors Fe(NO_3_)_3_·9H_2_O or Mg(NO_3_)_2_·6H_2_O.

### 2.3. Material characterizations

Fourier-transform infrared (FTIR) spectra were obtained using a Thermo SCIENTIFIC (NICOLET iS10) provided by Thermo Electron Corporation. The measurements were performed in the 500–3500 cm^−1^ range, with a material composed of 3 mg dilute in 250 mg of potassium bromide. The XRD (Bruker-D8) had employed to determine the phase purity of the synthesized nanocomposite aerogels. Scanning electron microscopy (Hitachi High-Tech) was utilized to obtain surface morphology images to evaluate composition. The porosity along with BET surface area of the materials made from MOF were determined using Fe–Mg adsorption/desorption analysis around 196 °C (BETtersizer).

## 3. Results analysis

### 3.1. SEM/XRD analysis

**Fig. 2:**
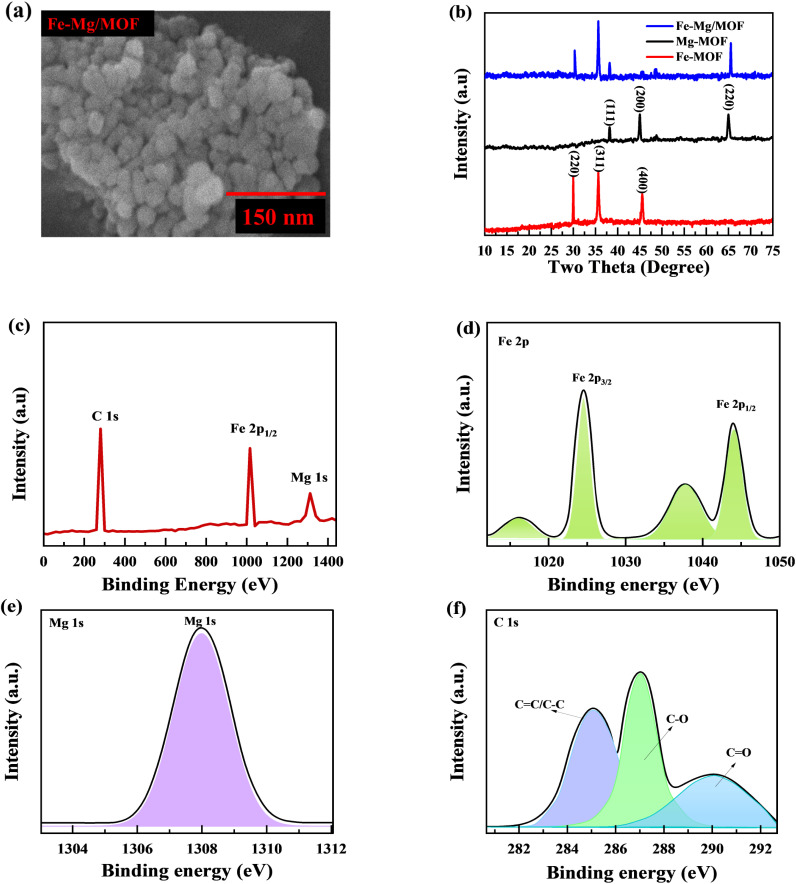
(a) Comprehensive SEM images exhibiting the morphology of MFM are presented. (b) Shows the Fm, MM and MfM XRD spectra. (c) Shows the XPS survey spectrum for MFM. (d–f) XPS spectrum of Fe 2p, Mg ls and C 1s, respectively.

Scanning electron microscopy is used to examine the structure and morphology of materials. Analyses of the samples' initial morphology show that the synthetic materials have a three-dimensional (3D) morphology (Fig. 2(a)). The acquired Fe-MOF was physically combined with the Mg and showed the morphologies of the doped material. These findings suggest that while the blending procedure also reduces particle size, the Mg and MOF produces a matrix that offers many active sites currently storing and accumulating charges.

The XRD analysis investigated the crystallization behaviors of FM, MM, and (1 : 1) MFM. The obtained results are depicted in Fig. 2(b). The XRD patterns of FM exhibit characteristic peaks at 2*θ* = 29.1, 36.2 and 45.9 with lattice points (220), (311) and (400), respectively.^30^

Similarly, the XRD patterns of MM display peaks at 2*θ* = 37.6, 44.3 and 65.2 with lattice points (111), (200) and (220) respectively.^31^ The findings confirm the successful formation of metal–organic frameworks characterized by excellent crystallinity and the absence of any oxide impurities. When the FM: MM ratio was 1 : 1, the XRD analysis revealed distinct diffraction patterns specific to MFM, indicating successful synthesis. Interestingly, the sample exhibited characteristic peaks corresponding to both MM and MF, confirming the incorporation of both components within the MFM structure. The intensity of the XRD peaks provided additional evidence for the coexistence of MM and FM in the synthesized MFM sample. These shifts are explained by the tractability effect of the FM arrangement, allowing for variations in the lattice parameters and crystal arrangement. Increasing the iron: magnesium ratio to 1 : 1 resulted in the combination of iron sort on the MM surface. These results demonstrate that Fe species have an impact on the MM structure as well as crystal size.

### 3.2. XPS analysis

The X-ray photoelectron spectroscopy (XPS) analysis was used to evaluate the chemical composition and determine the different electronic states. The combined appearance of MFM's FM, MM, and C components may be seen in the complete survey spectrum in Fig. 2. In Fig. 2(c), clear peak of Fe 2p were observed at 1025 eV while peaks for Mg 1s and C 1s were observed at 1307.3 and 288 eV, respectively. Fig. 2(d) displays the high-resolution spectrum of the Fe 2p region. The observed peaks at 1025.7 eV and 1045.3 eV correspond to Fe 2p_3/2_ and Mg 2p_1/2_, respectively, with a spin-energy separation of 19.6 eV. In Fig. 2(e) clear peaks are observed at 1308 eV, which can be attributed to the Mg 1s electronic configurations. While the C 1s spectra indicates the presence of three sharp peaks at 284.2, 286.5, and 290.3 eV which belongs near C=C/C–C, C–O, plus C=O, separately (Fig. 2(f)). These findings confirm the presence of FM, as reported in previous studies. These characteristics, namely the spin-energy separation and the presence of Fe^+^, indicate the presence of MFM. The analysis of reference spectra from standard samples confirmed that the Fe and Mg species in Fe–Mg/MOFs were both in the +2 valence state, which is consistent with the results obtained through XPS analysis.

### 3.3. Brunauer–Emmett–Teller analysis (BET)

**Fig. 3:**
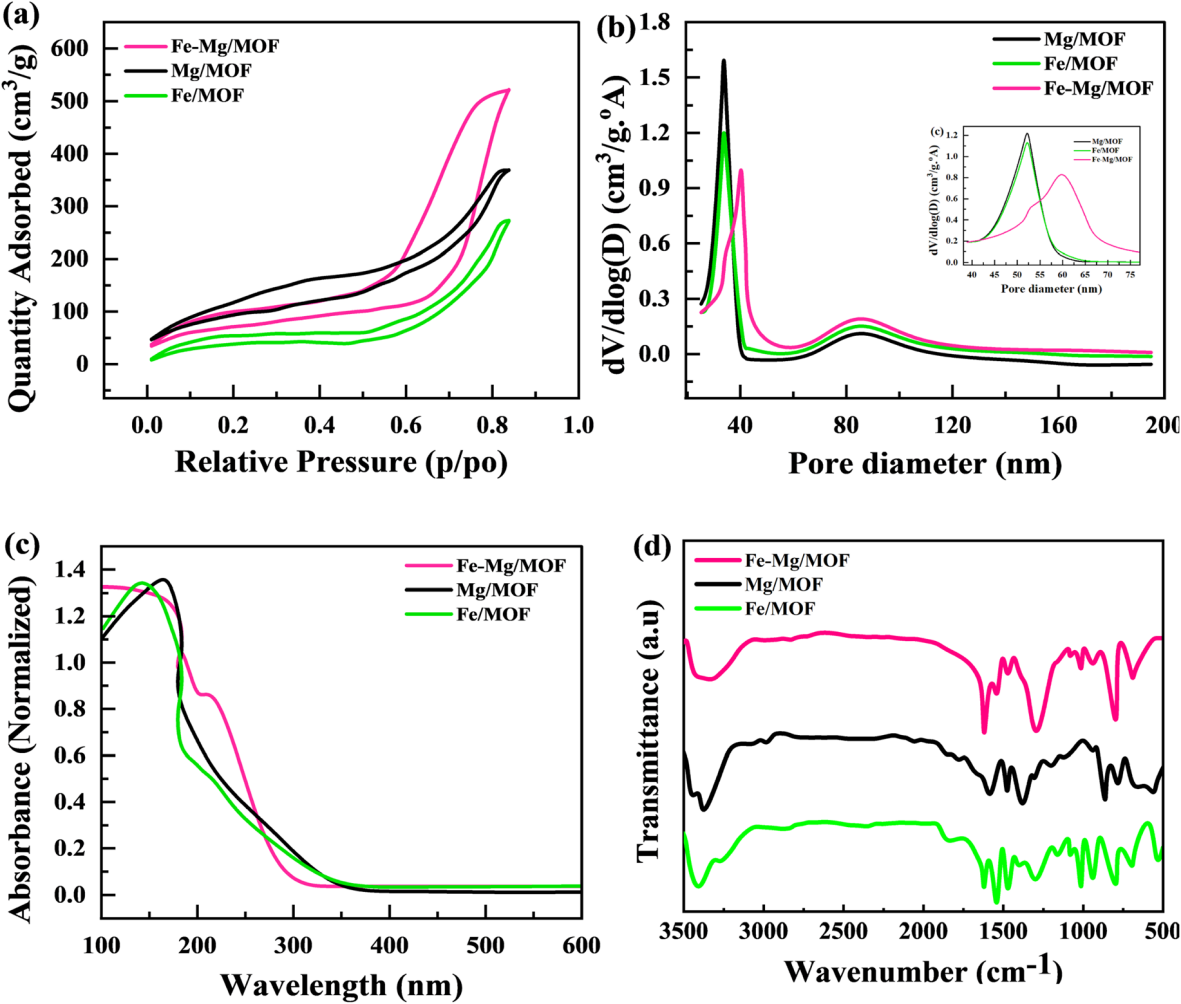
(a) Shows the BET nitrogen isotherm for FM, MM, and MFM. (b) BJH pore analysis for FM, MM, and MFM (c) absorption spectra of pure FM, MM and composite of MFM, respectively (d) FTIR spectra of the prepared FM, MM, and MFM.

Electrochemical reaction enhancement relies heavily on important factors such as surface area and pore size. The nitrogen adsorption–desorption isotherm of all the composites is depicted in Fig. 3(a). The MFM composite exhibits a BET-specific surface area of 324 m^2^ g^−1^. Incorporating MOF into the composites, such as MM, FM, and MFM, slightly increase their surface areas. The surface area of MM is 289 m^2^ g^−1^, FM is 238 m^2^ g^−1^, and MFM is 324 m^2^ g^−1^. The inclusion of MOF in the composites resulted in an augmentation of the surface area. Notably, MFM with a 2% MOF content demonstrated the highest BET surface area among all the composites. Fig. 3(b) represented the pore diameter for FM, MM, and MFM. This is advantageous as it increases the available surface area for electrolyte ion adsorption and provides more electroactive sites for redox reactions.

### 3.4. UV-vis spectrum

The UV-vis spectrum of FM, MM, and MFM in the 100–600 nm range were obtained to clarify their electronic characteristics (Fig. 3(c)). Fe-MOF showed separate peaks at 300 nm, indicating ligand–metal charge transfers, and 450 nm, indicating d–d transitions inside Fe centers. Mg-MOF exhibited a large absorption peak at around 280 nm, which was related with ligand-based π–π* transitions. The Fe–Mg/MOF hybrid spectrum exhibited a mix of Fe-MOF and Mg-MOF properties, with absorption peaks at 300 nm and 450 nm remaining, as well as the 280 nm peak ascribed to ligand-based transitions. These discoveries give information on the electrical structure and metal–ligand interactions in these materials, which is important for possible applications in a variety of fields.

### 3.5. FTIR analysis

In Fig. 3(d), the FTIR spectra of FM, MM, and MFM exhibit prominent characteristic peaks corresponding to the stretching vibrations of H–O–H bonds. Specifically, these peaks perceived by 812 cm^−1^ and 1672 cm^−1^ for MM and FM and are recognized near the strong C=O (carboxylic acid) extending vibration, indicating the presence terephthalic acid in metal organic frame-work. The vibration of stretching associated with C–O bonds is seen by the 1353 cm^−1^ peak. Furthermore, a peak 925 cm^−1^ suggests this substitution of aromatic ring. The findings show that the carboxyl groups in FM, MM, and MFM materials display deprotonation and share a structural framework built on deprotonated terephthalic acid.

### 3.6. TGA properties

**Fig. 4:**
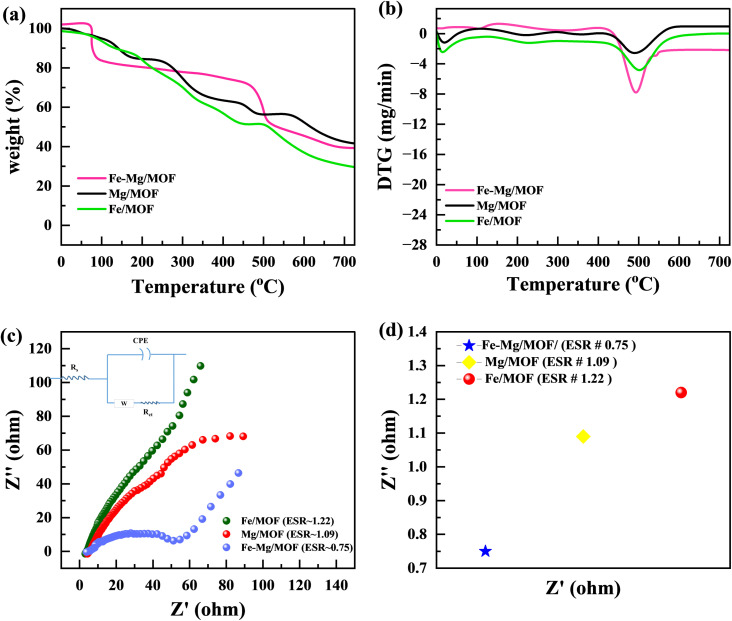
(a) TGA graph for FM, MM and MFM(b) DTG graph for FM, MM and FMM (c) Nyquist plot and enlarged view of a high-frequency region in the Nyquist plot (d) comparison of ESR value of FM, MM and MFM.

Fig. 4(a) and (b) illustrates the TGA (thermo gravimetric analysis) results of pure MM, FM, and MFM. The plot exhibits the weight loss profiles of each sample throughout the entire temperature range. The initial weight loss observed between 37 °C and 150 °C can be attributed to removing adsorbed water and eliminating solvents trapped within the material's pores. The second weight loss, occurring between 400 °C and around 500 °C, is associated with the structural collapse of the MOF framework. This temperature range leads to the organic ligand's decomposition and the framework structure's subsequent disintegration. The MFM compounds exhibited significantly enhanced stability, displaying a weight loss alternating after 400–700 °C, in contrast to this pure MM and FM.^32^ This property makes them particularly advantageous for utilization in supercapacitor applications.

## 4. Electrochemical characterizations

### 4.1. EIS analysis

According to Fig. 4(c), electrochemical impedance spectroscopy (EIS) was used to examine the charge transfer resistance with reaction kinetics. Lower equivalent series resistance (*R*_s_) as well as charge-transfer resistance (*R*_ct_) are characteristics of the MFM composites. In contrast to the MM electrodes, the FM electrode exhibits much lower *R*_s_ (1.39) and *R*_ct_ (0.78) values. This demonstrates the beneficial effect of Mg atom incorporation on improving the electron transport properties. In the low-frequency range, the MM sample has a very sharp slope of the line that is straight, showing a smaller diffusion resistance. This characteristic enables the efficient diffusion of ions in the electrolyte during the redox reaction, facilitating their movement.^33^ This strong interface facilitates rapid ion diffusion and minimizes internal resistance, leading to improved overall performance. The EIS analysis highlights that the combination of an oriented morphology structure in bimetallic MOFs can effectively minimize resistance related to charge transfer and ion diffusion. This improved design accelerates faradaic reactions, resulting in enhanced electrochemical performance of MOFs. The outstanding electrochemical characteristics of the MFM bimetallic complex could be attributed to the synergistic effect caused by the interaction of FM and MM, together with the unusual structural alignment. The combined impact of FM and MM appropriately results in improved structural stability, electrical conductivity, and enhanced electrochemical activity. Fig. 4(d) illustrates the Comparison of the ESR value of FM, MM and MFM.

### 4.2. Cyclic voltammetry (CV)

**Fig. 5:**
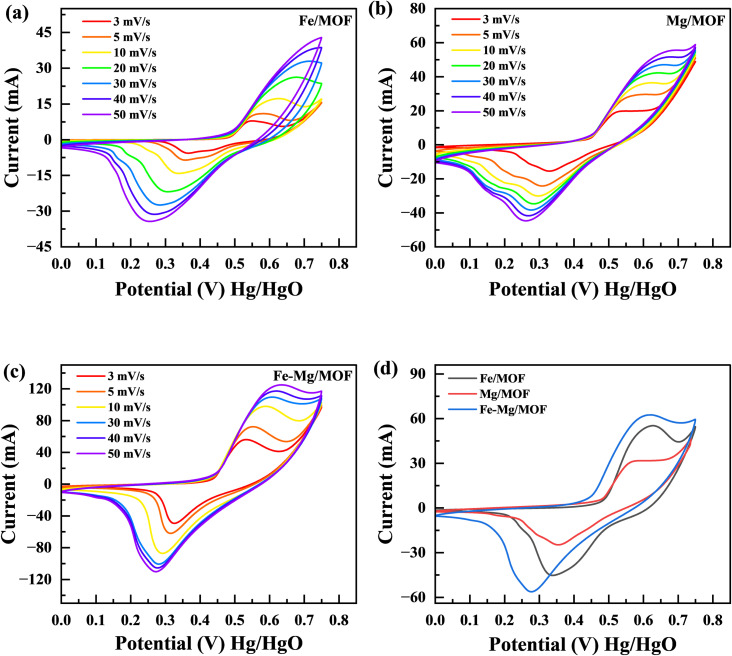
(a) CV curves for FM at various scan rates (3 mV s^−1^ to 50 mV s^−1^). (b) CV of MM at various scan rates (c) CV curves for the MFM (d) comparison of FM, MM and MFM at 5 mV s^−1^.

In order to examine the characteristics of the FM, MM, and MFM materials, cyclic voltammetry (CV) experiments were conducted. The CV curves in Fig. 5(a–c) represent the results obtained from the FM, MM, and MFM electrodes. The evaluations performed at different scan rates lie between 3 to 50 mV s^−1^ while maintaining a constant potential window of 0.75 V. Fig. 5(d) presents a comparison between MFM and its composites with FM and MM. The cyclic voltammetry curves of FM demonstrate distinct redox peaks, indicating faradaic electrochemical reactions and suggesting a battery nature. The maximum points in cyclic voltammetry peaks correspond to the oxidation as well as reduction processes of FM.^34^ These peaks provide valuable information about the nature of the faradaic reactions occurring within the material. When the oxidation and reduction peaks in a cyclic voltammetry (CV) curve are identical, it indicates a reversible electrochemical process. This suggests the electrode material can efficiently store and release charge without significant losses or side reactions. This implies that there may be some limited irreversibility or kinetic limitations associated with charge storage device. On the parallel side, if the oxidation as well as decrease peaks are distinctly different, it signifies an irreversible process. This indicates that the charge storage mechanism is delayed, and significant losses occur during the redox reactions. Assessing the performance and stability of electrode materials in different energy storage applications requires a thorough understanding of whether the electrochemical process is reversible or irreversible. This knowledge is important in determining the suitability and effectiveness of the material.^35^ The MFM shows characteristics of capacitive behaviour, leading to the absence of the material reduction peak commonly observed in the literature.^36,37^ The presence of MFM nanoparticles in large quantities hindered their involvement in the crystallization process of MFM. Consequently, these nanoparticles could aggregate and form conductive networks, facilitating conductivity.^38,39^ The addition of FM to MM leads to an increase in conductivity due to the enhanced movement of electrons and ions facilitated by electrostatic absorption/desorption processes. This phenomenon allows for faster and freer movement of charged particles within the material.^40^ Moreover, the MFM composites exhibited CV curves with prominent peak currents, indicating a higher charge storage capacity. The area covered by these CV curves is directly associated with the amount of charge stored by the material.^41^ Hence, the analysis of Fig. 5(b) and (c) reveals that the MM composite exhibits a significantly large area when related to FM separately. The large area observed in the graph signifies the MM maximum capacity and enhanced electroactive characteristics in contrast to FM when studied separately. The stability of the synthesized materials was demonstrated at higher scan rates by the form of the CV curves, which only slightly changed. These factors collectively determine the electrodes effectiveness in handling rapid charging and discharging processes. At higher scan rates, the specific capacity and output results tend to decrease due to a decrease during the reaction period among the electrolyte ions with the electrode. This reduced interaction time is often accompanied by an increase in kinetic irreversibility, resulting in decreased overall performance.^42^ The addition of MFM to the host material has been found to improve the electrochemical presentation of this electrode material. This improvement is supported by the Comparison of the cyclic voltammetery curves for FM, MM, and MFM composites, as depicted in Fig. 5(d). The specific capacity of the pure Fe-MOF is 797 C g^−1^ at 3 mV s^−1^ scan rate, under the same scan rate, MM composites has a greater specific capacity of 1075 C g^−1^. The MFM composites exhibit electroactive behavior and possess a large surface area, enabling an increased number of active sites for charge storage.

### 4.3. Galvanostatic charge/discharge (GCD)

**Fig. 6:**
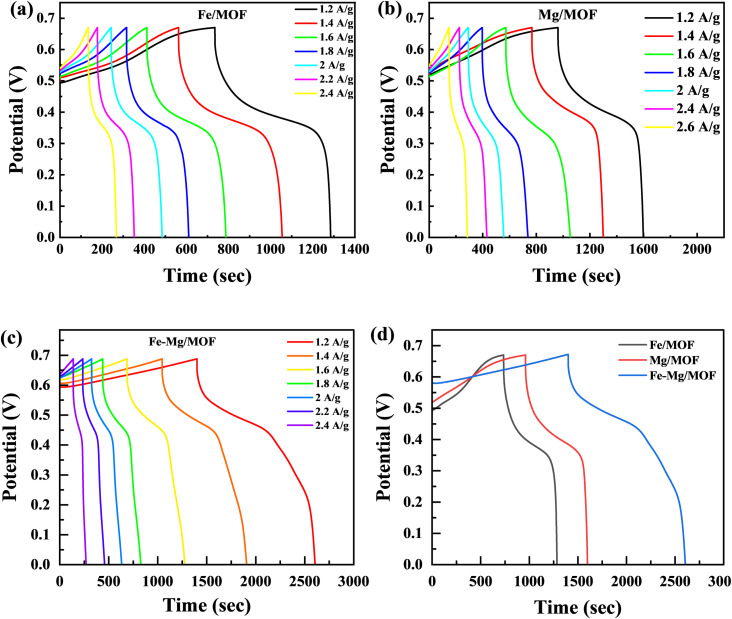
(a) GCD graphs for Fe-MOF at 1.2 A g^−1^ to 2.4 A g^−1^ (b) GCD curves for Mg-MOF (c) GCD curves for Fe–Mg/MOF at various current densities (d) comparison of FM, MM and MFM at 1.2 A g^−1^.

The charge storage capabilities of the FM, MM, and MFM electrodes were evaluated using the galvanostatic charge–discharge (GCD) method. Fig. 6 presents the galvanostatic charge/discharge investigation graph, which illustrates the electrochemical properties of the FM, MM, and MFM at various current densities. Upon analysis, it seen that FM electrode exhibited a comparatively low specific capacity.^43^ GCD analysis was performed on the FM composites, including MM and MFM, with a constant potential of 0.67 V across the current densities within the 1.2–2.6 A g^−1^ range. The GCD curves for MM and MFM are shown in Fig. 6(b) and (c), respectively. The results indicated that the discharging phase duration decreased as the current densities increased. The GCD curves of FM displayed the expected standard behaviour at varying current densities. However, the MM and MFM composites displayed significantly higher discharge peaks compared to FM. The synergistic reaction of MFM, which increases charge storage capacity, causes the discharge peak to increase. The presence of MFM enables a larger amount of charge to conduct more effectively, which leads to the observed amplification of the discharge peak. The GCD results support the CV findings and add to the evidence that the FM, MM, and MFM electrodes exhibit properties typical of battery-grade materials.

The FM, MM, and MFM electrodes exhibited remarkable redox reactions, the highest specific capacity with excellent electrochemical measurements. This was evident from its prolonged discharge period and parallel GCD curves, demonstrating its outstanding performance. Moreover, the electrode demonstrated negligible IR drop at high current densities, indicating low internal resistance and excellent conductivity.^44^ The GCD plots in Fig. 6(d) compare the FM, MM, and MFM electrodes within the potential range lies between the 0 to 0.7 V. However, the curves' non-linear segment suggests a redox reaction's involvement throughout the GCD process.^45^ In contrast to the inverted acute “*V*” pattern commonly observed in EDLC, the GCD analysis revealed that the host and doped materials (MM and MFM) exhibited predominant battery characteristics. Moreover, the time to discharge the MM and MFM electrodes significantly exceeded that of the pristine Fe-MOF electrode.^46^

**Fig. 7:**
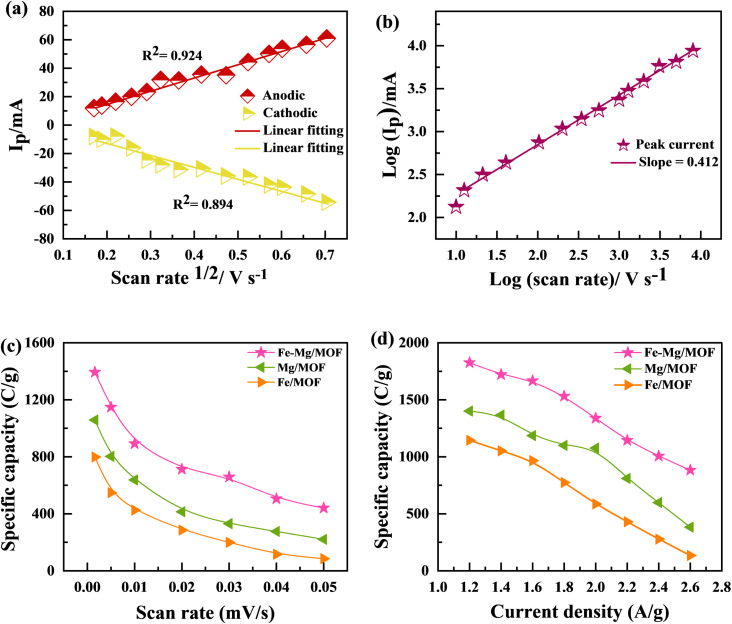
(a) Graphs between the anodic and cathodic peaks with scan rate 1/2 for Fe–Mg/MOF (b) graph between peak voltages and ln of scan rates (c) specific capacity was determined using CV with all three materials at different scan rates, (d) specific capacity measured using GCD as a function of current density.

Fig. 7(a) and (b) shows the MFM doped material peaks for anodic and cathodic processes, and the linear relationship achieved shows the material charge storage capability effectively. It observed that the value of *R* of anodic and cathodic peak current is close to value one, indicating that the material has reversible qualities. It is the basic criteria for battery-graded materials.^47^ By using the Randles servik equation,^48–51^ the reversible diffusion-controlled reaction for peak current can be calculated as1
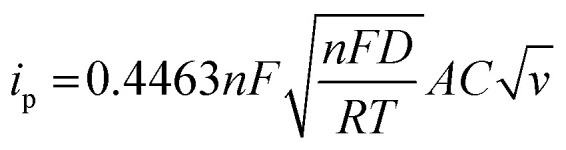


The value of peak current denoted by *i*_p_ in the above equation. The factors *n*, *F*, *T*, and *R* represent the number of electrons, Faradays constant, Kelvin temperature and gas constant, respectively. Factor *A* represents the surface area. Other factors *D* represents the diffusion coefficient, *C* shows the concentration in bulk form and *V* stands for voltammogram (at various) scan rates.^52,53^Fig. 7 also represents that process is diffusion controlled because according to the above equation peak current is proportional to 
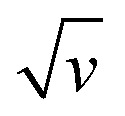
. Fig. 7(a) shows the value of slop around about 0. Confirming the pure process of the diffusion-controlled and demonstrating the materials is battery-grade. Fig. 7(b) graph indicates the linear behaviour within the voltage and ln of CV curves plotted in terms of scan rates that can be attributed to the implying MFM is a diffusion process. To evaluate, comparing the real slope of the straight line to the statistically predicted slope based on the diffusion-controlled mechanism.^49,50,54–56^

Fig. 7(c) and (d) present the specific capacity values obtained since the CV as well as GCD measurements. The data show that the specific capacity of FM calculated by CV was 797 C g^−1^, but the specified capacity calculated using GCD was 1144 C g^−1^. The MM sample exhibited a better-specific capacity of 1401 C g^−1^ at a current density of 1.2 A g^−1^ and 1057 C g^−1^ in CV at a scan rate of 3 mV s^−1^. The specific capacity of MFM was measured at 1393 C g^−1^ through CV and 1825 C g^−1^ through GCD analysis. These GCD results align with the earlier CV findings, indicating that MFM exhibits promising potential for utilization in supercapattery systems.

### 4.4. Supercapattery assembly

**Fig. 8:**
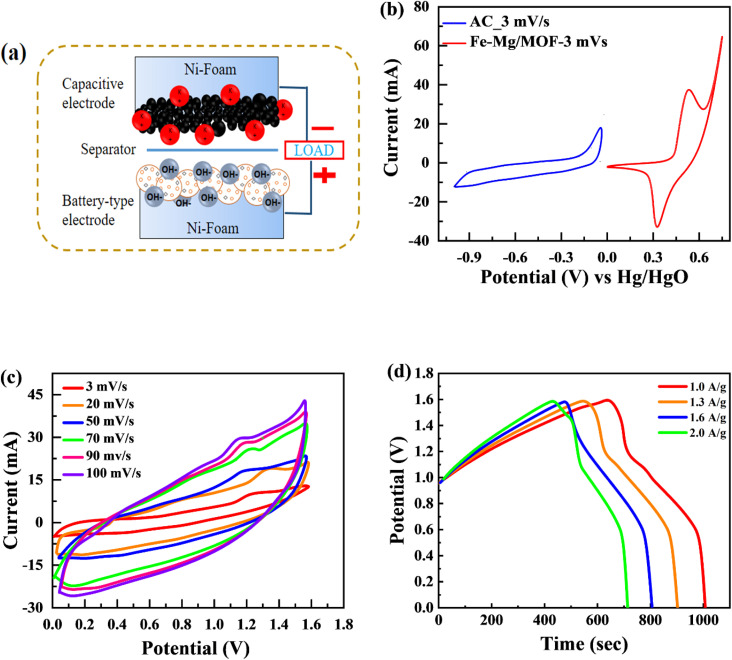
(a) Schematic of asymmetric supercapattery device (b) comparison of CV test between the AC and Fe–Mg/MOF (c) CV curves for a two-electrode assembly with scan speeds ranging from 3 to 100 mV s^−1^ utilizing a hybrid device. (d) GCD calculations for asymmetric device with a variable current density.

The MFM, which exhibited the best performance among the tested materials in the three-electrode arrangement, was further examined in a two-cell assembly. A schematic representation of the developed supercapattery device, utilizing MFM as the anode and activated carbon (AC) as the cathode, is shown in Fig. 8(a). The FM material was combined onto a nickel foam current collector and substrate, with a porous membrane separating it from the activated carbon. The device utilized activated carbon (AC) as its negative electrode, chosen specifically for its high porosity and conductivity. This unique combination enables enhanced ion absorption from the electrolyte.^57^Fig. 8(b) shows the applied potential window for activated carbon (AC) ranging from 0 V to −1 V, while the potential window for MFM spans from 0 V to 1.6 volts. As shown in Fig. 8(c), CV was used to evaluate the electrochemical behaviour of the supercapattery device based on MFM at various scan speeds (ranging from 3 to 100 mV s^−1^). The supercapattery device cyclic voltammogram peaks had quasi-rectangular forms, which became more distorted at higher potentials. This behavior can be attributed to two distinct energy storage mechanisms within the device, which causes various reactions. The deviation from the traditional rectangular shape of the CV curves at higher scan rates can be attributed to the limitation of ion transport during the redox process on the electrode surface. This restriction hinders the complete utilization of active sites, leading to a non-ideal shape.

Notably, the excellent rate capability of the supercapattery device (MFM) is evident from the retained shape of its cyclic voltammogram bends uniform on higher scan rates. Fig. 8(d) depicts the GCD curves obtained specifically for fabricated hybrid device utilizing MFM. These GCD curves exhibit a unique shape that blends triangular and humped profiles, resembling the characteristics observed in the cyclic voltammetry analysis. The supercapattery device (MFM) charging–discharging (GCD) curves are shown in Fig. 8(d) by different current densities. The potential window for the supercapattery device (MFM) is kept from (0–1.6) volts. The charging–discharging curves obtained at various current densities exhibited strikingly similar, indicating consistent performance. The minimal voltage drop across the internal resistance (IR) reflects a lower overall resistance. The capacity in question was calculated using the following the formula, which is based around the GCD graphs.^58^2
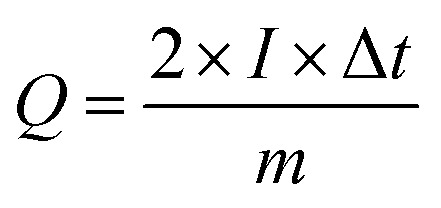


**Fig. 9:**
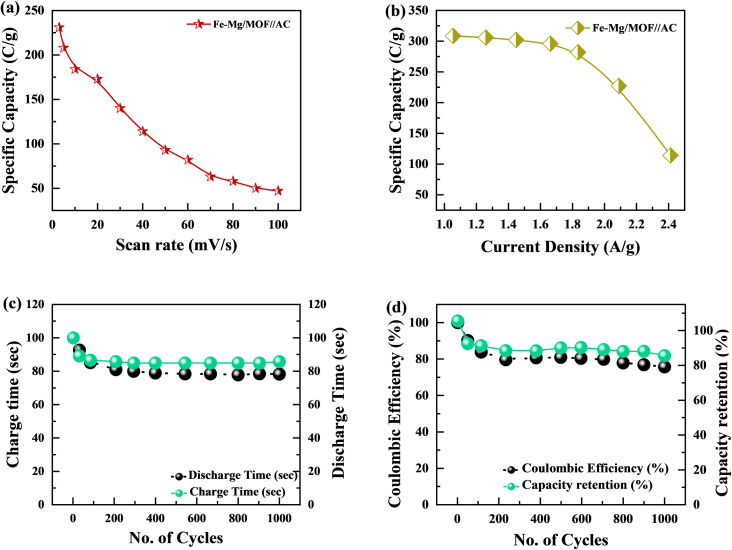
(a) Fe–Mg/MOF//AC specific capacity graph at various scan rates. (b) Specific capacity plotted against current density for Fe–Mg/MOF//AC. (c) This graph shows charge/discharge time *versus* 1000 cycles. (d) Coulombic efficiency and specific capacity retention after 1000 charging/discharging cycles.

**Fig. 10:**
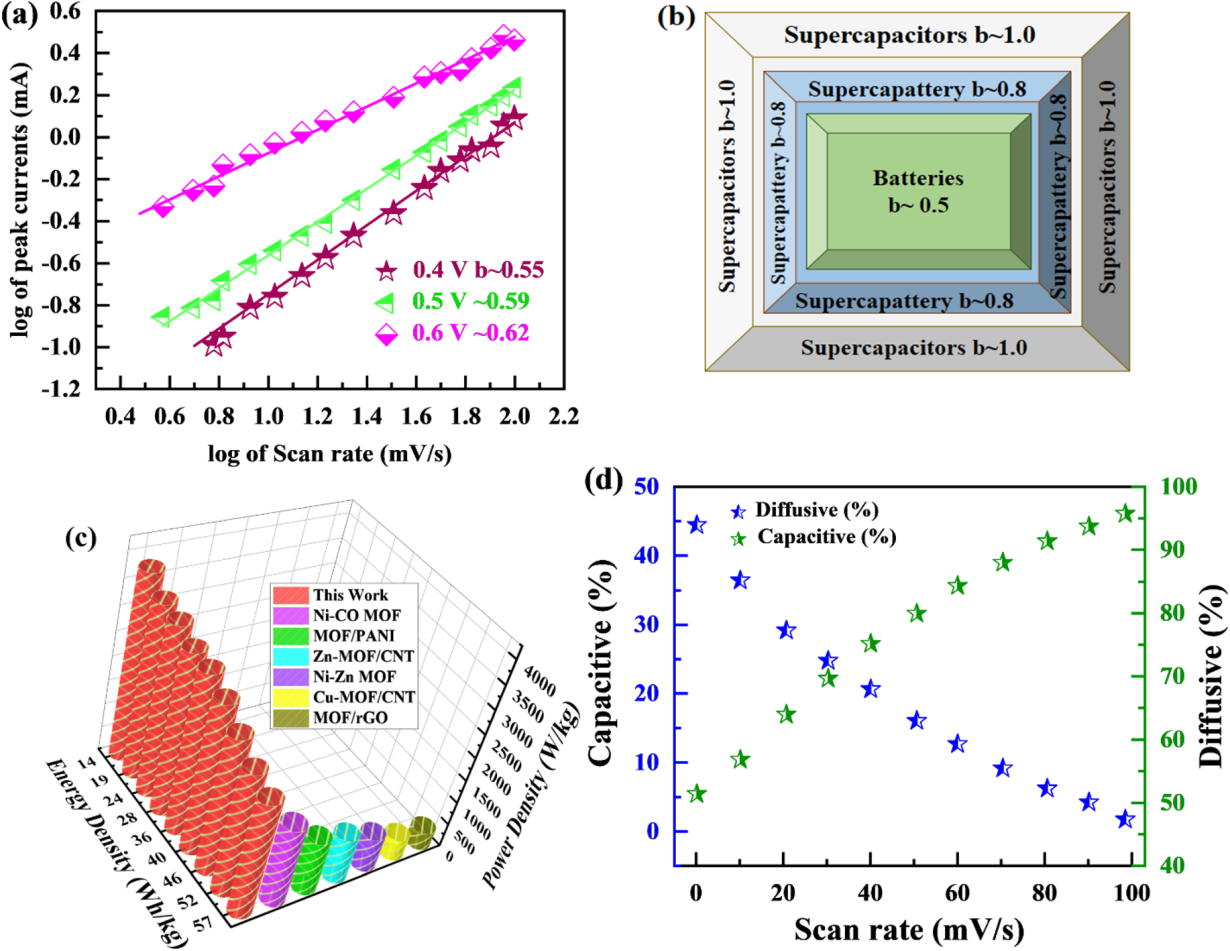
(a) The *b* value is calculated using linear regression of plots linking the logarithm of current to the logarithm of scan rate. (b) *b*-Fitting value used to identify various charge storage technologies (c) Ragone plot examining the relationship between power density and energy density. (d) Change in capacitive and diffusive contribution with scan rate.

The specific capacity is determined by eqn (2) using the discharge current (*I*), discharging period (*t*), and mass of active material placed on both substrates (*m*). Fig. 9(a) and (b), respectively, show the specific capacity obtained through the analysis of CV as well as GCD of the real device. The specific capacity of the supercapattery device (MFM//AC) was measured to be 231 C g^−1^ at a scan rate of 3 mV s^−1^ using the CV analysis. Furthermore, the GCD study revealed that the MFM//AC arrangement had a particular capacity of 301 C g^−1^ at a current density of 1 A g^−1^. The higher current density leads to a decrease in the capacity of the energy storage device. The limited duration primarily causes this decrease for ions to interact with the electrode material. Another important consideration for supercapattery devices is their cycle life, which refers to their ability to maintain performance over multiple charge–discharge cycles. To assess the durability of the MFM//AC configuration, 1000 charge–discharge cycles were performed at a current density of 4.0 A g^−1^. The results in Fig. 9(c) and (d) indicate that the maximum coulombic efficiency achieved during these cycles was 81.8%. Furthermore, the capacity retention after 1000 cycles was 85%. These results show that the MFM//AC supercapattery device exhibits excellent cycle stability and capacity retention. Fig. 10(a) depicts the relationship between the scan rate and peak current logarithms, which were utilized to calculate the *b*-fitting values. When the *b*-values fall between 0 and 0.5, it indicates battery-like behavior. *b*-Values within the range of 0.8 to 1.0 suggest supercapacitor-like characteristics.

The MFM//AC supercapattery device *b*-values, shown in Fig. 10(b), support being classified as a supercapattery. The device which merges the qualities of batteries and supercapacitors has a determined *b*-value that is in the range of 0.5 to 0.8. The MFM//AC device has distinctive charge storage behavior, making it appropriate for various energy storage applications, as this research clearly demonstrates.^59^ Energy density and power density are essential factors that determine the performance of an energy storage device. Energy density refers to the amount of energy that can be stored in a given volume or mass of the device. It indicates how much energy the device can hold; higher energy density means more energy can be stored. The energy and power density were calculated using the following equations.3
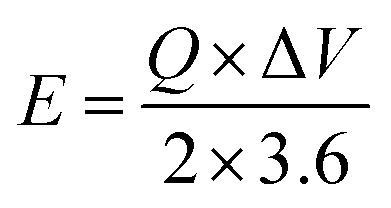
4
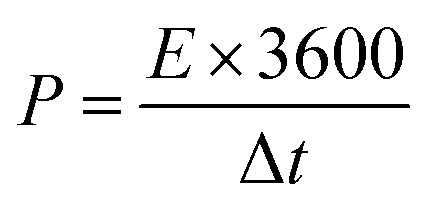


The equations mentioned above utilize the following variables: Δ*V* for the potential window, *Q* for the specific capacity, the symbol *E* stands for energy density and the symbol Δ*t* for the discharge time, *P* represents the power density. Then specific energy density of the supercapattery device (MFM//AC) was calculated to be 57 W h kg^−1^ at a power density of 2393 W kg^−1^. These values indicate the amount of energy that can be stored and the rate at which it can be delivered by the device, respectively. A comparison of the results obtained in this study with the literature is presented in Fig. 10(c). These values highlight the device's superior performance in its ability to deliver power rapidly while retaining a reasonable energy storage capacity.

**Fig. 11:**
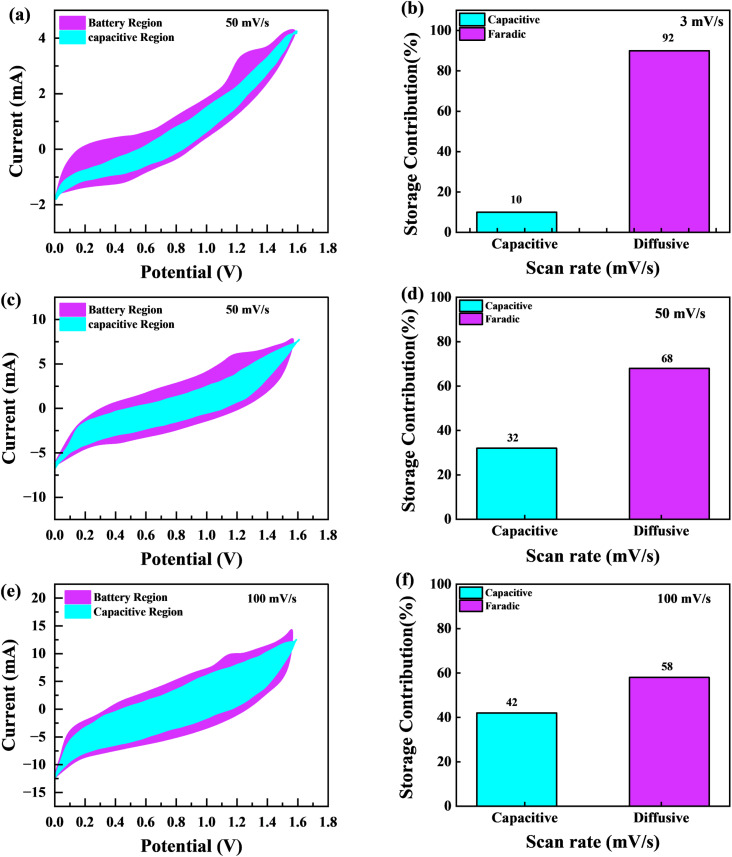
(a, c and e) CV shows the capacity contribution as a percentage of the total contribution at 3, 30, 50 and 100 mV s^−1^ (b, d and f); the ratio of faradaic *versus* non-faradaic contribution is shown in a bar plot.

Fig. 11(a), (c) and (e) graphically depicts current contributions generated at different scan rates for 3, 50, and 100 mV s^−1^ through capacitive and diffusive regulated processes. The tinted green part in the graph represents the capacitive contribution in the cyclic voltammetry graph. For different scan rates, such as 3, 50 and 100 mV s^−1^, Fig. 11(b), (d) and (f) shows identical bar graphs illustrating the capacitive along with diffusive percentages. The capacitive involvement, which is 10%, is rather minimal compared to the diffusive impact, which is 90% slower at the minimum scan rate of 3 mV s^−1^ gradually, capacitance contribution rises as the scan rate rises, but the diffusive value falls. At a maximum scan rate of 100 mV s^−1^, the capacitive contribution is 42%, while the diffusive contribution is measured at 58%. This shows that with the slower scan rate, there is a greater diffusive contribution for the entire device capacity, providing the positive electrode enough time ample the redox process. These findings support our assertion on the supercapattery, the technology that combines the advantages of supercapacitors and batteries.

## 5. Hydrogen evolution reaction

**Fig. 12:**
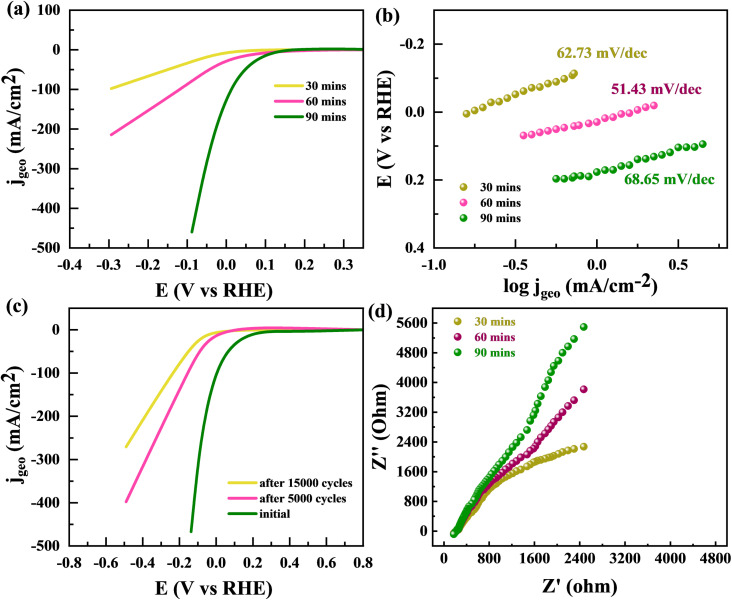
(a) HER polarisation curves are shown for MFM after 30 min, 60 min, and 90 min. (b) Tefel plots for MFM after 30 min, 1 h, and 2 h (c) LSV curve for MFM after 5000 and 15 000 CV repitations. (d) EIS spectrum for MFM after 30 min, 1 h, and 2 h.

By utilizing the benefits offered by large, concerned with edge-sites particular volume of a sponge-like structure, the edge-oriented Fe/Mg-MOF can be employed as an electrochemical hydrogen evolution reaction catalyst. The capability to use material as an electrode to measure its electrochemical characteristics is another benefit of edge oriented Fe/Mg-MOF structure developed upon flexible Mg substrates. This is due to the fact that additives like glassy carbon aren't required. The usual cathodic polarization curves and related Tafel plots support the fascinating hydrogen evolution reaction activity of edge-oriented Fe/Mg-MOF in three different annealing times (made upon 300 °C about 30, 60, and 90 min). HER onsets of the Fe/Mg-MOF films are between 150 and 200 mV, the same as what has been reported for nano-scale Fe/Mg-MOF.^60,61^ At an over potential of 220 mV, highest IR-corrected kinetic current density normalised to geometrical area is 18.6 mA cm^−2^. This is higher than the less edge-oriented Fe/Mg-MOF structure made with other annealing times (30 and 90 min) (Fig. 12(a)). The slopes of the Tafel diagrams for the Fe/Mg-MOF (60 min annealing) films are between 51.43 mV dec^−1^ which is better then at 30 min (62.73 mV dec^−1^) and 90 min (68.65 mV dec^−1^) as indicated in Fig. 12(b). This shows that the Volmer-reaction is happening, which is such process that turns proton into H-atoms that stick to the sponge like Fe/Mg-MOF surface. It is a factor in the HER mechanism that controls the rate of the reaction.^62^ Compared to edge-terminated Fe/Mg-MOF compact films and porous Fe/Mg-MOF films that have been reported recently,^63,64^ the sponge-like Fe/Mg-MOF films with edge-oriented nanostructures have high kinetic current densities as well as low Tafel slopes. Most of the better HER performance can be attributed to the open and porous shapes, which make it easier to expose more active edge sites and give more ways for ions and masses to move. Fig. 12(c) showed the stability performance of Fe/Mg-MOF after 5000 and 15 000 CV repitations. The Fe/Mg-MOF demonstrates a remarkable ability to sustain its HER (Hydrogen Evolution Reaction) efficiency, preserving a significant 80% efficiency after enduring a tough series of 10 000 stability experiments, as shown in Fig. 12(c). Despite being subjected to 20 000 stability tests, the material's HER performance remained commendably good at 74%. In Fig. 12(d), we investigate the electrochemical properties of the Fe/Mg-MOF using EIS (Electrochemical Impedance Spectroscopy). The EIS spectra produced clearly depict the changing impedance properties of the Fe/Mg-MOF throughout time intervals of 30 minutes, 1 hour, and 2 hours. As the time span widens, a recognizable trend emerges from the data: the impedance of the Fe/Mg-MOF increases gradually but consistently. This observed increase in impedance highlights the material's developing electrochemical dynamics, indicating an important interaction between the surface of the Fe/Mg-MOF and the surrounding electrolyte. These findings not only emphasize the Fe/Mg-MOF's outstanding stability and durability in long-term HER applications, but also shed insight on the complicated interplay among its electrochemical characteristics and the passage of time. Such discoveries pave the way for an improved comprehension of the Fe/Mg-MOF's performance and bring up possibilities for future tuning in catalytic applications.

## 6. Conclusion

This research highlights using a unique class of bi-metallic Fe–Mg/MOF for supercapattery devices. A hydrothermal method was used for the synthesis of the required substances. Electrochemical characterization was done in the three and two-electrode assembly to examine the charge storage application. This bi-metallic Fe–Mg/MOF exhibits remarkable electrochemical characteristics in a three-electrode setup with specific capacities 1825 C g^−1^ at 1 A g^−1^. The synthesized Fe–Mg/MOF material is combined with activated carbon to study the real-time application to form a supercapattery. The durability of the supercapattery was also assessed over 1000 galvanostatic charging/discharging cycles which demonstrated remarkable 85% capacity retention. Additionally, Fe–Mg/MOF when tested in hydrogen evolution reaction (HER) showed outstanding electrocatalytic performance by demonstrating Tafel slope of 51.43 mV dec^−1^ This Fe–Mg/MOF-based material is a hopeful contender for high-performance supercapattery devices.

## Conflicts of interest

There are no conflicts to declare.

## Supplementary Material

RA-013-D3RA04279K-s001
