# Supplementary material for: A bimetallic Fe–Mg MOF with a dual role as an electrode in asymmetric supercapacitors and an efficient electrocatalyst for hydrogen evolution reaction (HER)
Source: RSC Adv. 2023 Sep 5;13(38):26528–43. doi: 10.1039/d3ra04279k (PMC10477833; doi:10.1039/d3ra04279k)
Supplement: RA-013-D3RA04279K-s001 [file RA-013-D3RA04279K-s001.pdf]

Electronic Supplementary Material (ESI) for RSC Advances.
This journal is © The Royal Society of Chemistry 2023

Bimetallic Fe-Mg MOF: A Dual Role as an Electrode in Assymetric Supercapacitors and an Efficient Electrocatalyst for Hydrogen Evolution Reaction (HER)

Asma Zaka[1], Muhammad Waqas Iqbal[1]*, Amir Muhammad Afzal[1], Haseebul Hassan[1], Hira Rafique[1], Saikh Mohammad Wabaidur[2], Ahmed M Tawfeek[2], Eshan Elahi[3]

[1]*Department of Physics, Riphah International University, Campus Lahore, Pakistan*

[2]*Chemistry Department, College of Science, King Saud University, Riyadh 11451, Saudi Arabia*

[3] *Department of Physics, Sejong university, Republic of Korea*

E-mail: waqas.iqbal@riphah.edu.pk,

Supplementary part

According to figure S1 in the supplementary section, the specific capacities of Fe/MOF and Mg/MOF were determined from CV at various scan rates and from GCD at various current densities. Supercapattery assemblies were also created in the supplementary section using Fe-Mg/MOF. This device used Fe-Mg/MOF as the positive electrodes and activated carbon as the negative electrode. The 1 M KOH solution was used as the electrolyte for all calculations. Figure S2 (a) shown the experimental deposition of activated material for two electrode assembly and figure S2 (b-c) displayed a three-electrode array as well as a two-electrode arrangement. The working material Fe-Mg/MOF and activated carbon (AC) electrodes have been combined utilizing a porous material as a separator, as can be seen in Figure S2 (d).

Figure S3 (a-b) displays the specific capacities obtained from CV graphs for the Fe/MOF //AC and Mg/MOF//AC devices respectively. This graph shows that the maximum specific capacity of Fe/MOF and activated carbon (Fe/MOF //AC) device was 178 C/g, and the specific capacity

calculated using GCD for the Fe-Mg/MOF //AC was 267 C/g. (c) Specific capacity was determined using CV of Mg/MOF at different scan rates was 283 C/g, (b) Specific capacity calculated by using GCD of Mg/MOF as a function of current density was 347 C/g.

The Fe/MOF//AC device has been shown to retain an 83 percent specific capacity retention and a 72 percent columbic efficiency after being subjected to 1000 charging/discharging cycles during durability testing, as illustrated in Figure S4 (a-b). Figure (c-d) showed the Mg/MOF//AC with 75% capacity retention and 84% columbic efficiency after the 1000 charge/discharge cycles.

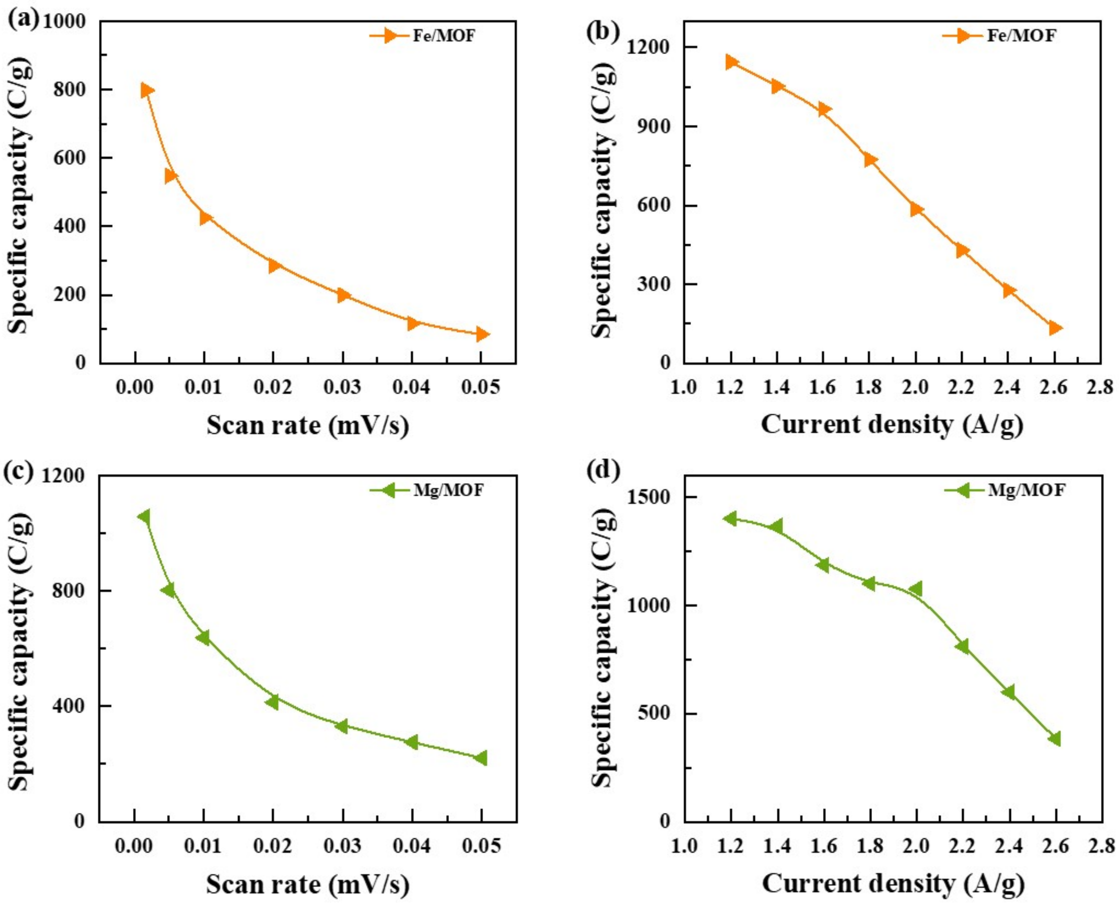

Figure S1. (a) Specific capacity was determined using CV of Fe/MOF at different scan rates, (b) Specific capacity calculated by using GCD of Fe/MOF at various current density (c) Specific capacity was determined using CV of Mg/MOF at different scan rates, (b) Specific capacity calculated by using GCD of Mg/MOF as a function of current density.

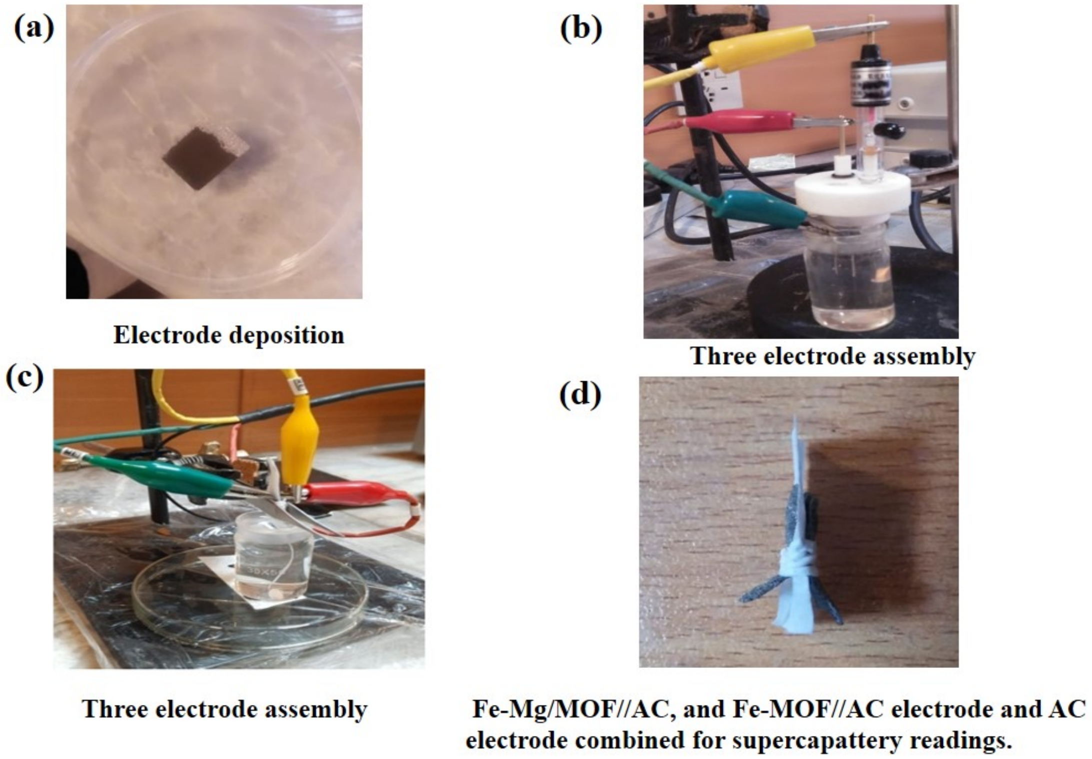

Figure S2. (a) The experimental deposition of activated material for two electrode assembly (b) An image of three-electrode assemblies was captured during the experiment (c) During the experiment, an image of two-electrode assemblies was taken. (d) The Fe-Mg/MOF//AC

Fe/MOF//AC and Mg/MOF//AC electrodes were attached to the AC electrode for supercapattery measurements.

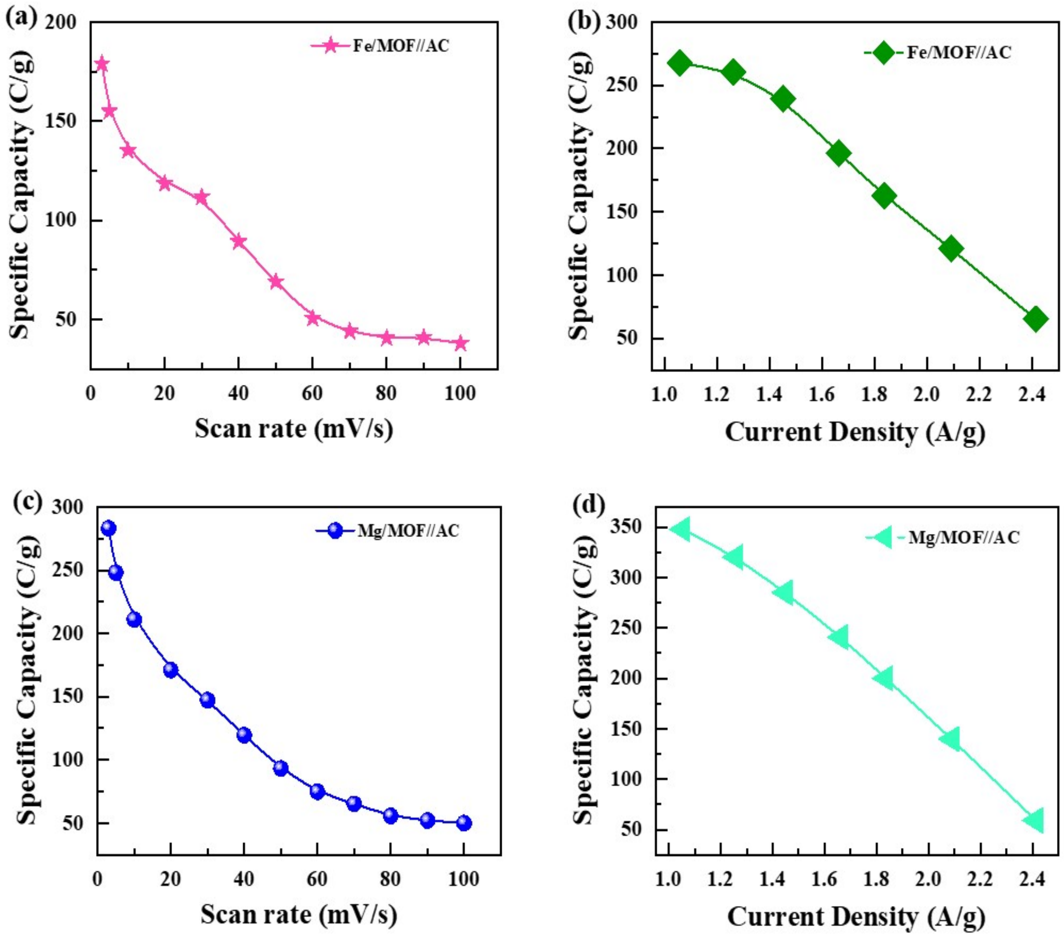

Figure S3 (a) CV at various scan rates were used to calculate the specific capacitance of the Fe/MOF/CNT//AC device. (d) GCD trends at different current densities were used to determine the specific capacitance of the Fe/MOF/CNT//AC device (c) CV at various scan rates were used to calculate the specific capacitance of the Mg/MOF/CNT//AC device (d) GCD trends at different

current densities were used to determine the specific capacitance of the Mg/MOF/CNT//AC device.

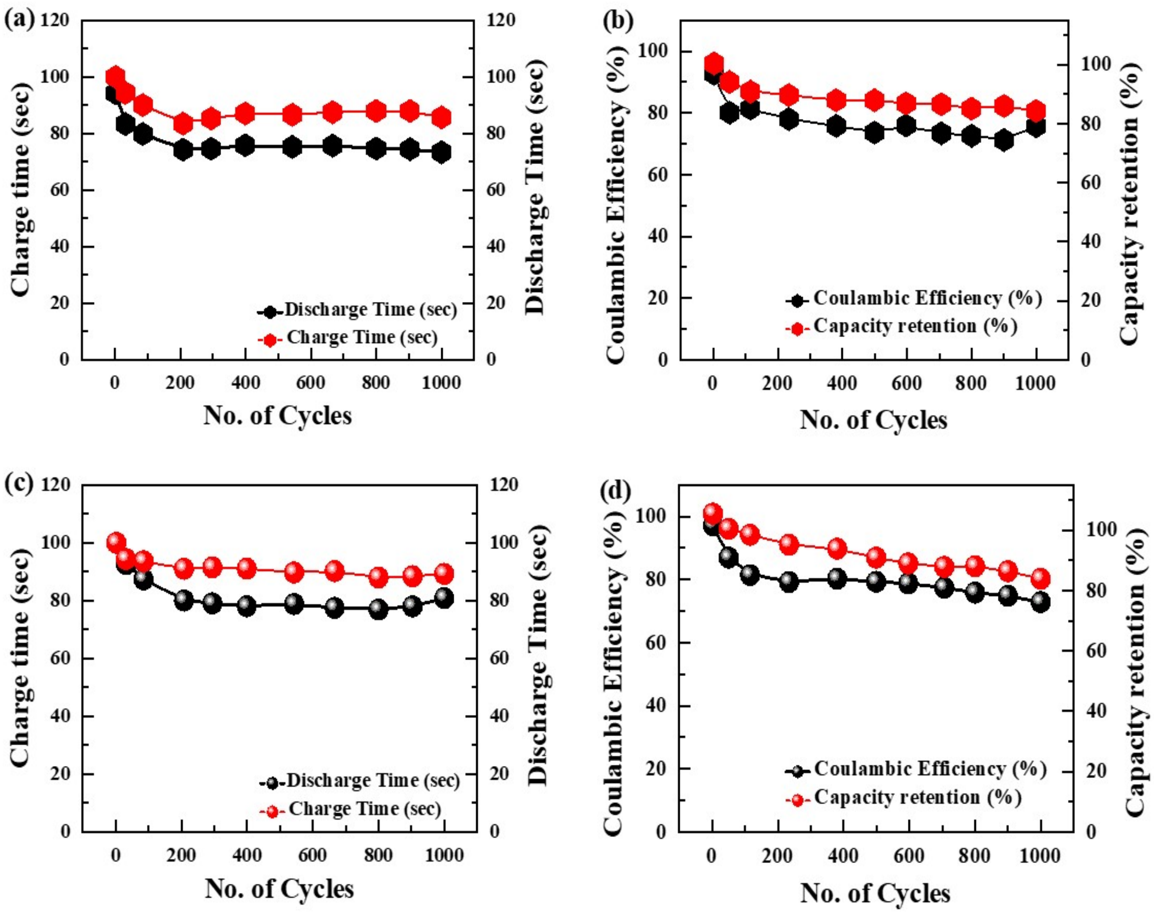

Figure S4 (a) This graph shows Fe/MOF//AC charge/discharge time versus 1000 cycles (b) Columbia efficiency and specific capacity retention Fe/MOF//AC after 1000 charging/discharging cycles (c) This graph shows Mg/MOF//AC charge/discharge time versus 1000 cycles. (d)

Columbia efficiency and specific capacity retention Mg/MOF//AC after 1000 charging/discharging cycles.
